# Achievement of renal anemia KDIGO targets by two different clinical strategies – a European hemodialysis multicenter analysis

**DOI:** 10.1186/s12882-018-1196-7

**Published:** 2019-01-07

**Authors:** Maciej Drozdz, André Weigert, Fatima Silva, João Frazão, Abdulkareem Alsuwaida, Mahesh Krishnan, Werner Kleophas, Szymon Brzosko, Fredrik K. Johansson, Stefan H. Jacobson

**Affiliations:** 1 DaVita Poland, Krakow, Poland; 2 DaVita Portugal, Lisbon, Portugal; 3 Nephrology Department, Hospital Santa Cruz, Carnaxide, Portugal; 4 School of Medicine, University of Lisbon, Lisbon, Portugal; 5 Department of Nephrology, São João Hospital Center, Porto, Portugal; 6 School of Medicine, University of Porto, Porto, Portugal; 7 DaVita Saudi Arabia, Riyadh, Saudi Arabia; 8 King Saud University, Riyadh, Saudi Arabia; 9 DaVita, Inc, Denver, CO USA; 10 DaVita Germany, Dusseldorf, Germany; 11 Clinic for Nephrology, Heinrich-Heine –University, Dusseldorf, Germany; 12 1st Department of Nephrology and Transplantation, Medical University of Bialystok, Bialystok, Poland; 13 DaVita Poland, Bialystok, Poland; 14 Unit for Medical Statistics, Department of Clinical Sciences, Karolinska Institutet, Danderyd University Hospital, Stockholm, Sweden; 15 Division of Nephrology, Department of Clinical Sciences, Karolinska Institutet, Danderyd University Hospital, Stockholm, Sweden

**Keywords:** Anemia, Erythropoiesis-stimulating agent, Ferritin, Hemodialysis, Hemoglobin, Iron, Mortality, TSAT

## Abstract

**Background:**

The optimal treatment algorithm for iron therapy and the use of erythropoiesis-stimulating agents (ESA) in anemic hemodialysis (HD) patients has not been established. Hemoglobin (Hb) target levels can be achieved through more frequent intravenous (IV) iron use with lower ESA dose, or with less iron dosing but higher ESA. ESA therapy to correct anemia may result in severe arterial and venous thrombotic complications and the evidence base evaluating hard clinical outcomes related to the use of IV iron is sparse.

**Methods:**

A total of 1247 maintenance HD patients from 12 dialysis centers in Portugal (*n* = 730) and Poland (*n* = 517) were considered. We assessed achievement of KDIGO renal anemia targets with focus on treatment strategies, which typically differ between countries. In Poland the use and dose of IV iron was 35–72% higher than that in Portugal (*p* <  0.001) during three consecutive months; use and dose of ESA was 61% higher in Portugal (5034 vs 3133 IU (adjusted)/week, *p* <  0.001).

**Results:**

Mean Hb concentration was similar (11.0 vs 11.0 g/dL) in patients treated in both countries and the proportion of patients within KDIGO anemia target was 69.5% in Poland vs 65.8% in Portugal (NS). Ferritin and TSAT levels and the proportion of patients with TSAT > 20 and > 50% were both significantly higher in patients in Poland (88.8 and 14.6%) than in Portugal (76.3 and 5.7% respectively, p <  0.001). Significantly more patients in Poland had a ferritin concentration > 800 μg/L (35.6%) compared to Portugal (15.8%, p <  0.001). The ESA resistance index (ERI) was significantly higher in patients treated in Portugal (p <  0.001). Correlation analyses showed confounding by treatment indication in unadjusted models. Multiple and logistic regression analyses showed that with ferritin within KDIGO recommended range of 200–800 μg/L the odds for Hb within guidelines increased significantly. Annual gross mortality was 16% in Poland and 13% in Portugal (NS); there were no differences in cause-specific mortality.

**Conclusions:**

Administration of high doses of IV iron in routine clinical HD practice may not be associated with considerable harm. However, large randomized controlled trials are needed to provide absolute evidence of iron safety.

## Background

Anemia in patients on hemodialysis (HD) significantly impairs quality of life. Several previous studies have shown that more severe anemia is associated with higher risk of left ventricular hypertrophy, cardiovascular events, transfusion-related infections, hospitalization and death [1, 2]. Anemia management in HD patients has focused on the use of erythropoiesis-stimulating agents (ESA) and iron therapy, but, following concerns regarding the safety of using high doses of ESA to meet aggressive Hb targets that were raised by clinical trials such as NHS [3], CREATE [4], CHOIR [5, 6] and TREAT [7],interest in the use of intravenous (IV) iron therapy has increased [8, 9].

Many patients on HD are in negative iron balance because of poor appetite and reduced dietary intake of iron, reduced absorption from the gut, and increased gastrointestinal iron losses. Hemodialysis- and laboratory test-related blood loss vary substantially over time and among patients and are also influenced by anticoagulant and antiplatelet therapy [10, 11].

Currently, serum iron, serum ferritin and transferrin saturation (TSAT) are commonly assessed as markers of iron metabolism in clinical practice. However, these indicators are far from optimal as they can be influenced by malnutrition and inflammation, both of which are common in patients on HD [12, 13]. IV iron administration is often required to attain iron repletion and increases in Hb values in such patients, and also to optimize the treatment response to ESA. However, concerns have been raised regarding the safety of iron compounds due to the possibility of iron overload, oxidative stress, cardiovascular disease, hypersensitivity reactions, and exacerbation of infection [14, 15].

In addition, ESA therapy in patients on HD may result in many adverse clinical consequences, especially stroke, venous thromboembolic disease, and vascular access thrombosis [16] and some patients do not respond adequately to ESA [17, 18].

Currently, the evidence base relating to the use of IV iron in combination with ESA is limited, and the effects of IV iron on clinical outcomes, like death and major health events remain uncertain.

The Kidney Disease Improving Global Outcomes (KDIGO) clinical practice guideline on anemia management [19] proposes a broader use of iron therapy in HD patients, including higher values of TSAT and ferritin at which iron therapy should be initiated or discontinued. Since the publication of the guideline, there have been significant changes in anemia management practices in in many countries, with a notable shift towards lower Hb levels, lower ESA doses and greater use of IV iron [8, 9]. Data from the Dialysis Outcomes and Practice Patterns Study (DOPPS) have shown progressive increases in serum ferritin in recent years, with nearly 40% of the HD population in the US having ferritin levels greater than 800 ng/mL. Similar trends have been observed in some European countries [8].

In the present European multicenter analysis, we compared clinical practices and strategies to achieve the KDIGO targets for renal anemia in a large cohort of patients on HD in 12 DaVita centers in Portugal and Poland as part of our continuous quality improvement efforts. In addition, annual mortality was analyzed and compared between countries and centers.

## Methods

We included 1247 patients on maintenance HD from DaVita Portugal (5 dialysis centers, *n* = 730, mean age 69 ± 14 years (SD), 40% females) and DaVita Poland (7 dialysis centers, *n* = 517, mean age 67 ± 15 years, 47% females) in an analysis of the achievement of KDIGO renal anemia targets and focused on treatment strategies, which by tradition and socioeconomic factors differ in the two countries (Table 1 and Table 2). The patient cohort includes all prevalent HD patients treated at all DaVita facilities in Portugal and Poland, and thus represent a “real world” clinical observation from two regions in Europe that previously haven’t been included in the global DOPPS analysis.

**Table 1:** Demographics and laboratory profile

mean (SD)	All	Portugal	Poland	p*
Number of patients	1247	730	517	–
Age (years)	68 (14)	69 (14)	67 (15)	< 0.01
Time on dialysis (months)	60.7 (58.5)	66.1 (63.7)	53.2 (49.4)	< 0.001
Weight post dialysis (kg)	70.2 (22.1)	68.2 (13.8)	74.3 (29.8)	< 0.001
BMI (kg/m^2^)	26.0 (7.8)	25.2 (4.7)	27.1 (10.7)	< 0.001
Hemoglobin (g/dL)	11.0 (1.3)	11.0 (1.3)	11.0 (1.3)	N.S.
TSAT (%)	31.3 (14.5)	28.5 (12.9)	35.3 (15.5)	< 0.001
Ferritin (μg/L)	605.4 (491.5)	497.9 (344.3)	757.1 (613.5)	< 0.001
Weekly dose of ESA (corrected) (U)	4306 (5134)	5154 (6077)	3133 (3068)	< 0.001
Iron dose (mg per month 1)	176 (172)	143 (176)	246 (141)	< 0.001
Iron dose (mg per month 2)	164 (164)	147 (173)	198 (141)	< 0.001
Iron dose (mg per month 3)	176 (180)	151 (187)	230 (150)	< 0.001
spKt/V	1.8 (0.4)	2.0 (0.4)	1.6 (0.3)	< 0.001
URR (%)	77.5 (8.1)	79.9 (6.7)	74.2 (8.7)	< 0.001
Weekly treatment time (min)	729 (56)	724 (53)	737 (55)	< 0.001
Treated blood volume (L/kg)	1.34 (0.32)	1.45 (0.35)	1.20 (0.24)	< 0.001
IDBWG (kg)	2.3 (1.7)	1.9 (1.7)	2.9 (1.5)	< 0.001
Albumin (g/L)	40.7 (3.7)	40.4 (3.4)	41.2 (3.6)	N.S.
Calcium (mg/dL)	8.8 (0.7)	8.9 (0.6)	8.7 (0.8)	< 0.001
Phosphorus (mg/dL)	4.4 (1.4)	4.1 (1.2)	4.7 (1.5)	< 0.001
Intact PTH (pg/mL)	563 (531.5)	561 (518.0)	565 (551.2)	N.S.

^*^Students t-test between hemodialysis facilities in countries

*N.S*. = Not significant

*Abbreviations*: *BMI* body mass index, *TSAT* transferrin saturation, *ESA* erythropoiesis-stimulating agents, *spKt/V* single pool Kt/V, *URR* urea reduction ratio, *IDBWG* interdialytic body weight gain, *PTH* parathyroid hormone

**Table 2:** Patient characteristics

	All	Portugal	Poland	p*
	(%)	(%)	(%)	
Hemoglobin 10–12 g/dL	67.3	65.8	69.5	N.S.
Hemoglobin > 12 g/dL	15.2	15.3	15.1	N.S.
Ferritin ≥200 μg/L	84.4	82.9	86.6	N.S.
Ferritin ≥800 μg/L	24.0	15.8	35.6	< 0.001
TSAT ≥20%	81.5	76.3	88.8	< 0.001
TSAT ≥50%	9.4	5.7	14.6	< 0.001
Treatment with ESA	76	78	74	0.056
Type of ESA				N.S.
Darbepoietin	24	22	26	
Epoetin beta	76	78	74	
Route of ESA administration				< 0.001
Intravenous	64.5	99.8	11.5	
Subcutaneous	35.5	0.2	88.5	
ESA administration, frequency				< 0.001
No ESA	23.6	21.7	26.3	
Every other week	0.4	0.7	0	
Once per week	15.0	2.1	33.2	
Twice per week	15.9	11.1	22.6	
Three times per week	45.1	64.5	18.0	
Dialysis modality				< 0.001
HDF	46.6	91.4	0	
HD	53.4	8.6	100	
spKt/V > 1.2	97.3	98.5	95.6	0.003
URR ≥ 70%	90.8	95.4	84.4	< 0.001
Treatment time ≥ 12 h/week	93.8	94.0	93.6	N.S.
Prescribed Qb ≥ 300 mL/min	94.1	97.4	89.4	< 0.001
Treated blood volume ≥ 1 L/kg body weight	90.0	95.7	81.9	< 0.001
CVC (%)	16.5	14.5	19.3	0.026
AV Fistula (%)	76.8	77.1	76.4	N.S.
S-Albumin ≥35 g/L	89.7	95.4	81.8	< 0.001
Charlson comorbidity index				< 0.001
< 7	44.5	39.1	52.1	
7–12	51.9	56.7	45.2	
> 12	3.6	4.3	2.7	
Diabetes mellitus	26	30	25	N.S.
Phosphorus ≤5.5 mg/dL	82.0	87.5	74.2	< 0.001
Calcium ≤10.2 mg/dL	98.1	98.0	98.2	N.S.
PTH 150–600 pg/mL	53.9	52.1	56.4	N.S.

^*^ Chi-2 analysis between countries

Demographic data and information on practices were collected during the same month in all patients in both countries. Monthly iron doses were recorded during three consecutive months in both regions. Blood samples were collected monthly or quarterly in accordance with international clinical guidelines (European best practice guidelines, KDIGO guidelines) and results were reviewed by the national chief medical officer and discussed with the European chief medical officer monthly.

The most common cause of end-stage renal disease (ESRD) in both Portugal (42%) and Poland (35%) was classified as “unknown” or “other”. Diabetes mellitus, nephrosclerosis and glomerulonephritis were the causes of ESRD in 30, 10 and 11% of HD patients in Portugal; the corresponding percentages were 25, 13 and 20% for patients in Poland (*p* <  0.001).

All laboratory analyses were performed at local laboratories according to validated and recommended routine procedures. Intact parathyroid hormone (PTH) was measured. spKt/V = single pool Kt/V. The correction factor for darbepoietin to epoetin doses was 250. The erythropoiesis-stimulating agent resistance index (ERI) was defined as the weight-adjusted weekly ESA dose divided by the hemoglobin value [IU/kg/(g/dl)].

The study was approved by the Regional Ethics committee in Stockholm, located at the Karolinska Institutet, Stockholm, Sweden. All clinical and laboratory patient data were sent deidentified from the respective country and all statistical analyses were performed by SHJ and FKJ at the Department of Clinical Sciences at the Karolinska Institutet, Stockholm, Sweden.

### Statistical analyses

Statistical analyses were done using IBM SPSS Statistics version 25 at the Unit for medical statistics at the Karolinska Institutet, Stockholm, Sweden. All values are presented as mean or median and comparisons between countries were performed using Student’s t-test and Chi-2 analysis as appropriate. Correlations were done using Pearson correlation and multiple regression and logistic regression analyses were used to adjust for covariates. Results are given as odds ratio with additional *p*-value and beta coefficient with *p*-value. Since we do not have information on the exact date of death for each individual patient in this study, we were unable to assess hazard ratios using Cox regression analysis. A *p*-value < 0.05 was considered statistically significant.

## Results

### Demographic and laboratory profiles

Table 1 and Table 2 show demographic and clinical mean (SD) values and category (%) data for all patients as well as for patients from the respective country. HD patients treated at centers in Portugal were significantly older (69 vs 67 years), had longer dialysis vintage (66 vs 53 months), lower body weight and BMI than the corresponding patients treated in Poland (Table 1). A high Charlson comorbidity index, 7–12 or > 12 was significantly more frequent among HD patients at facilities in Portugal than in patients in Poland (*p* <  0.001, Table 2). Serum albumin was 40 (3) g/L and 41 (4) g/L in units in Portugal and Poland, respectively (NS), but the proportion of patients with a serum albumin ≥35 g/L was significantly higher in Portugal (95%) than in Poland (82%, *p* <  0.001, Table 2). Serum phosphorus was significantly higher (*p* <  0.001) and the proportion of patients with serum phosphorus ≤5.5 mg/dL significantly lower (*p* <  0.001) in patients in Poland than in Portugal, but there were no significant differences in intact PTH (Table 1 and Table 2).

### Prescription of hemodialysis

In facilities in Portugal, almost all patients had hemodiafiltration (HDF) while in Poland 91% had conventional hemodialysis (100% high flux membranes) and 9% had HDF (*p* <  0.001, Table 2). An AV fistula (AVF) was used as a vascular access in 77% of patients in centers in Portugal and in 76% of patients in Poland (NS, Table 2). spKt/V > 1.2 and a urea reduction ratio (URR) ≥ 70% was achieved in almost all patients, but more often so in Portuguese patients that in Polish patients (*p* = 0.003 and *p* <  0.001 respectively, Table 2). Mean weekly treatment time was 724 (53) min in Portugal and 737 (55) min in Poland (*p* <  0.001) and the prescribed dialysis blood flow rate (Qb) and the corresponding treated blood volume per dialysis session were both significantly higher in patients in facilities in Portugal than in patients in Poland (*p* <  0.001 for both comparisons, Table 1 and Table 2).

### Renal anemia

**Fig. 1:**
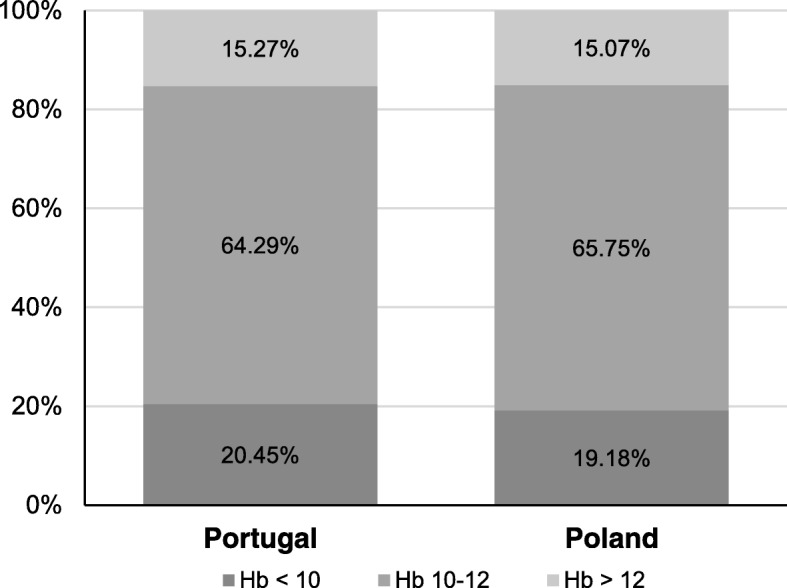
Proportion of patients above, within and below KDIGO Hb targets in HD patients in centers in Portugal and Poland

The mean Hb was similar in patients treated in units in Portugal (11.0 (1.3) g/dL) and in Poland (11.0 (1.3) g/dL) and the proportion of patients within the recommended KDIGO anemia target of Hb 10–12 g/dL was also similar (66 and 70% respectively, Table 1, Table 2 and Fig. 1). 78.3% of patients in Portugal received ESA compared to 73.7% in Poland (NS, Table 2). The weekly ESA dose corrected for epoetin/darbepoetin (× 250) was significantly higher 5154 (6077) U in HD patients in Portugal as compared to those in Poland (3133 (3068) U, *p* <  0.001). The route of administration of ESA was also different with more IV administration in Portugal (Table 2). There were no significant differences in the prescription of different types of ESAs in the two countries. ERI was 6.23 IU/kg/(g/dL) in all hemodialysis patients and significantly higher in patients in Portugal (7.53) compared to patients in Poland (4.42; *p* <  0.001). Multiple regression analysis of the ERI index using backward selection adjusting for age, dialysis vintage, Kt/V, serum albumin, Charlson comorbidity index and country (Poland vs Portugal) showed that the country had the highest impact on changes in ERI (beta value − 3.462, *p* <  0.001).

**Fig. 2:**
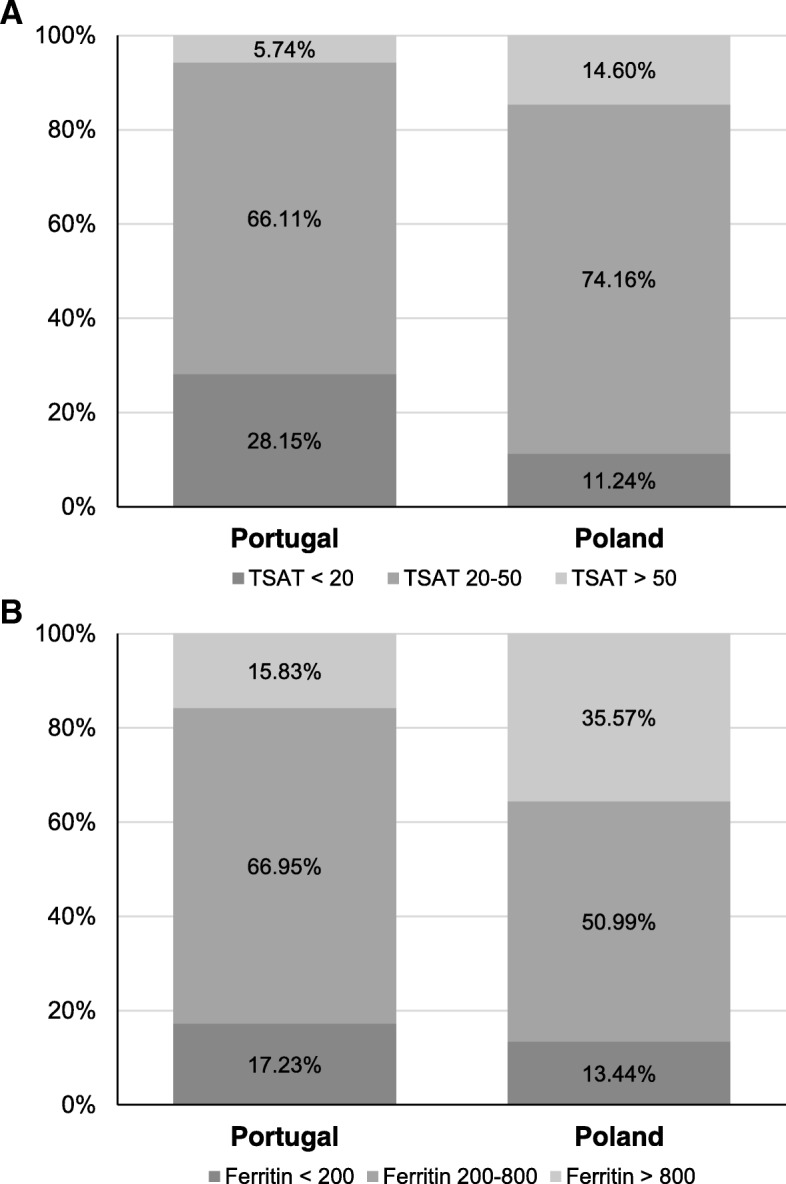
Proportion of patients above, within and below KDIGO TSAT (**a**) and ferritin (**b**) targets in HD patients in centers in Portugal and Poland

In dialysis centers in Poland the use and monthly doses of IV iron were 35–72% higher than in Portugal during three consecutive months (*p* <  0.001, Table 1). TSAT and the percentage of patients with TSAT ≥20% and TSAT > 50% were all significantly higher (*p* <  0.001 for all three comparisons) in patients treated in Poland (Table 1, Table 2 and Fig. 2) compared to those in Portugal. Furthermore, serum ferritin as well as the percentage of patients with serum ferritin > 800 μg/L were significantly higher (*p* <  0.001 for both comparisons) in patients treated in Poland compared to those in Portugal (Table 1, Table 2 and Fig. 2).

We also analyzed the achievement of Hb targets in relation to gender in all 1247 patients (70% of females were within Hb target vs 65% of males, NS) as well as in the respective country (Portugal, female vs male, 70% vs 63%, NS and Poland 71% vs 68%, NS).

In relation to vascular access use, 68% of all patients with an AVF had Hb between 10 and 12 g/dL as compared to 64% of all patients with a central venous catheter (CVC, NS). Comparisons between countries showed that 68% vs 69% of patients with an AVF in units in Poland and Portugal, respectively, achieved Hb within KDIGO targets (NS). However, among patients with a CVC significantly more patients in Poland (74%) had Hb between 10 and 12 g/dL compared to corresponding patients in facilities in Portugal (48%, *p* <  0.001).

### Correlations, multiple regression and logistic regression analysis

Correlation analysis (Table 3) showed significant negative correlations between Hb and ferritin in all patients (Pearson correlation coefficient, *r* = − 0.13, *p* <  0.01), in patients in units in Portugal (*r* = − 0.22, *p* <  0.01) but not in Poland (NS). There were no significant correlations between Hb and TSAT in all patients or in the respective country. Furthermore, we observed significant negative correlations between Hb and the corrected (epoietin/darbepoietin) weekly dose of ESA in all patients (*r* = − 0.27, *p* <  0.01) as well as in patients in centers in Portugal (*r* = − 0.25, *p* <  0.01) and Poland (*r* = − 0.38, *p* <  0.01). Taken together these findings are examples of confounding by treatment indication. Ferritin is also a marker of systemic inflammation and patients who are hyporesponsive to ESA—for example, due to malnutrition or inflammation—are often prescribed higher doses of ESA to achieve treatment targets in clinical practice.

**Table 3:** Analyses of correlation (r = Pearson correlation coefficient) between achieved Hb and ferritin, TSAT and weekly dose of ESA in all patients and in Portugal and Poland respectively

	All	Portugal	Poland
Hb vs Ferritin	−0.125**	−0.222**	−0.078
Hb vs TSAT	0.041	0.028	0.046
Hb vs corrected weekly dose of ESA	−0.271**	− 0.250**	− 0.384**

^**^ *p* < 0.01

Multiple regression (Table 4) of the effect on Hb (beta value) including age, gender, BMI, TSAT, ferritin, albumin and PTH in the analysis showed that ferritin had the strongest influence on Hb and that the effect on Hb is − 0.004 (*p* <  0.001). Subsequent logistic regression showed an odds ratio of 1.521 (*p* <  0.001) for ferritin 200–800 μg/L to achieve Hb within target (10–12 g/dL). This association was strongest in patients treated at the centers in Portugal (odds ratio 1.865, *p* <  0.001, Table 4) but not so in patients in Poland. Thus, with ferritin within KDIGO recommended ranges of 200–800 μg/L the odds for an Hb within guidelines increases significantly.

**Table 4:** Multiple (a) and logistic (b) regression analysis of effects on Hb g/dL and the recommended Hb target of 10–12 g/dL

	All	Portugal	Poland
(a) Multiple regression analysis of effect of the respective variable on Hb in all patients and in Portugal and Poland, respectively			
	Beta value (*p*)	Beta value (*p*)	Beta value (*p*)
Age	0.002 (NS)	0.002 (NS)	0.006 (NS)
Gender	−0.166 (0.024)	−0.196 (0.035)	− 0.068 (NS)
BMI	0.006 (NS)	−0.007 (NS)	0.009 (0.029)
TSAT	0.008 (0.002)	0.011 (0.005)	0.006 (NS)
Ferritin	−0.0004 (< 0.001)	−0.001 (< 0.001)	0.0002 (< 0.001)
Albumin	0.005 (0.018)	0.070 (< 0.001)	0.004 (< 0.001)
PTH	0.0003 (< 0.001)	0.0002 (0.026)	0.0003 (< 0.001)
(b) Logistic regression analysis with Hb 10–12 g/dL as effect measure			
	Odds ratio (*p*)	Odds ratio (*p*)	Odds ratio (*p*)
Age (years)	1.005 (N.S.)	0.991 (N.S.)	1.027 (< 0.001)
Gender (M/F)	1.267 (N.S.)	1.337 (N.S.)	1.098 (N.S.)
BMI (kg/m^2^)	0.995 (N.S.)	0.987 (N.S.)	0.988 (N.S.)
Albumin (> 35 g/dL)	1.444 (N.S.)	1.314 (0.042)	1.498 (N.S.)
TSAT (20%–50%)	1.438 (0.008)	1.314 (N.S.)	1.498 (N.S.)
Ferritin (200–800 μg/L)	1.521 (0.001)	1.865 (< 0.001)	1.341 (N.S.)
PTH (150–600 pg/mL)	0.921 (N.S.)	0.967 (N.S.)	0.806 (N.S.)

### Mortality

**Fig. 3:**
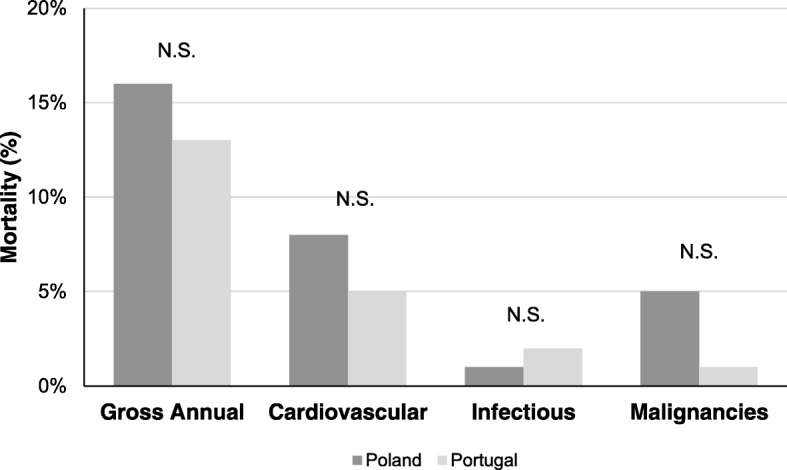
Gross annual mortality and cause specific mortality (%) in patients treated in Portugal and Poland

We also studied gross and cause-specific mortality over 1 year in the respective country and at all dialysis centers within countries. One-year gross mortality was 16% in Poland and 13% in Portugal (NS); there were no significant differences in one-year mortality between centers in the respective country. There were also no differences in cause specific mortality (cardio- and cerebrovascular, infectious and malignancy) between countries (Fig. 3).

## Discussion

The most important clinical finding of this large multicenter European cross-sectional analysis of all prevalent patients treated at 12 hemodialysis units in Portugal and Poland is that the KDIGO targets for the treatment of renal anemia in hemodialysis were achieved in both countries, but through different clinical practices and strategies. The Hb concentration was similar between countries and the proportion of patients within KDIGO target range was 69.5% in Poland and 65.8% in Portugal. The prescribed monthly doses of iron were 35–72% higher in dialysis facilities in Poland as compared to those in Portugal (*p* <  0.001). Consequently, the ferritin and TSAT levels and the proportion of HD patients with a TSAT > 20 and > 50% were both significantly higher in HD patients in centers in Poland (88.8 and 14.6%) than in Portugal (76.3 and 5.7%, respectively). In addition, significantly more patients in units in Poland had a ferritin concentration > 800 μg/L (35.6%) compared to those in Portugal (15.8%). By contrast, the prescribed ESA doses were 65% higher in HD patients in dialysis facilities in Portugal (5154 U) than in Poland (3133 U). Multiple and logistic regression analyses showed that with ferritin within KDIGO recommended ranges of 200–800 μg/L the odds for Hb within target range increases significantly. Taken together, both treatment traditions and socioeconomic factors seem to play a role in how anemia is managed in these facilities.

The optimal treatment algorithm for iron therapy and ESA use in anemic HD patients has so far not been established. Many would agree that in clinical practice one should avoid prescribing disproportionately high ESA or iron doses to achieve internationally recommended Hb targets, particularly in patients with significant comorbidity and/or ESA resistance. There were some differences in patient demographics and practices between patients in the clinics in Poland and Portugal in the present study. HD patients in facilities in Portugal were older and had a longer dialysis vintage, while more patients had diabetes and a high Charlson comorbidity index, all indicating a larger comorbidity burden than in patients in units in Poland. Despite this, significantly more patients in centers in Portugal had a serum albumin > 35 g/L and the use of a CVC was less frequent than in units in Poland.

The Optimal Renal Anemia Management Assessment (ORAMA) trial, published a decade ago [20], prospectively examined the impact of implementing anemia guidelines on outcomes relating to the management of renal anemia [21]. Results of the study showed that hemoglobin values were significantly higher in HD patients in Western Europe than those in Eastern Europe. While these findings suggested that Eastern European patients were being treated to lower Hb target levels at the time of the study, to our knowledge there are no recent European updates on this issue.

Several observational studies have shown that anemia in HD patients is associated with an increased risk of cerebro- and cardiovascular events, hospitalization and mortality [1, 2, 22]. However, given that renal anemia often is a marker of comorbidity, a cause-effect relationship has not yet been proven. Concerns about correcting anemia with high doses of ESA in CKD have been raised [5–7]. The TREAT study showed an increased cardiovascular risk with ESA use at high Hb values [7]. Randomization to the high Hb target group had a no effect on the primary composite cardiovascular end point, but a higher risk of venous and arterial thromboembolic events and stroke was observed, the latter unrelated to mean arterial blood pressure. Recently, a study of a large cohort of incident Spanish HD patients revealed a dose-dependent relationship between ESA dose and mortality risk: higher ESA doses were associated with a higher mortality, despite adjustments for potential confounders, including Hb and covariates related to ESA hyporesponsiveness [23]. In the present study, patients in Portugal had significantly higher ESA doses, but one-year gross and cause-specific mortality was similar (13%) to that in Poland (16%).

In the present study, both treatment strategies—focusing on either higher ESA doses (+ 65%) with modest doses of iron, or a higher prescription of iron (+ 35%–72% per month) with less ESA requirement—resulted in Hb values within KDIGO guideline targets in the majority of patients. ESA hyporesponsiveness is common among dialysis patients, affecting up to 10% of patients with renal disease receiving ESA [24]. Iron deficiency, infection/inflammation, and inadequate dialysis are the most common cause of hyporesponsiveness [24]. In the present study, the ERI was significantly higher in patients treated in Portugal compared to patients treated in Poland. A recent large observational study showed that patients treated with HDF had significantly lower ERI, but that this study was limited to patients treated with intravenous erythropoietin [25]. The type of ESA prescribed was similar in our study, but in facilities in Portugal ESA was more frequently administered intravenously. The impact of route of administration on ESA dose requirements and responsiveness have been the subject of numerous studies, but so far no consensus has been reached. A recent evaluation of 24,957 patients receiving HD between 2011 and 2014 revealed no difference in ESA dose between subcutaneous (SC) and IV administration, despite an increasing proportion of patients receiving SC [23]. Furthermore, higher doses of ESA were associated with an increased risk of hospital admissions and mortality, with no differences between SC and IV routes of administration [26].

There is general consensus that iron therapy should be prescribed for HD patients who are iron deficient and may increase Hb values, delay the need for ESA therapy, and also reduce ESA requirements once therapy is initiated [27]. Both TSAT and ferritin have their shortcomings as measures with which to assess iron status and guide iron therapy in HD patients. TSAT and ferritin values generally considered to be indicative of iron deficiency are higher for patients on HD than for the general population due to the presence of inflammation [28]. There is also debate on the safety and efficacy of IV iron therapy, particularly for those patients who already have high serum ferritin levels. Infusion of iron in HD patients may overwhelm the capacity of the iron binding proteins, resulting in the presence of free iron in the circulation and/or to increase iron stores. Indeed, previous studies have shown that after IV iron injection in HD patients there is a transient increase in oxidative stress, as evidenced by an increase in plasma lipid peroxidation and oxidative modification of proteins [15]. Currently, there is no clear evidence to support a specific “safe” upper limit for serum ferritin levels but KDIGO guidelines recommend not exceeding a TSAT of 30% and serum ferritin of 500 ng/mL during iron therapy [19].

Large controlled clinical trials assessing the impact of more aggressive iron therapy on clinical outcomes have so far not been conducted, and observational studies have yielded inconsistent results. A study by Miskulin et al. [29] assessed 14,078 patients initiating dialysis in the US between 2003 and 2008 and showed that receipt of ≤1050 mg intravenous iron in 3 months or 2100 mg in 6 months was not associated with increased risk of all-cause, cardiovascular, or infection-related mortality. The same group [30] observed no consistent association between IV iron dose and risk for all-cause, cardiovascular, or infectious hospitalizations, even among patients receiving iron doses exceeding 2100 mg over a period of 6 months. A new multicenter observational study of the safety of IV iron supplementation in incident HD patients showed that larger iron doses infused over time were not associated with mortality, after adjusting for potential confounding factors [31]. Furthermore, a recent systematic review and meta-analysis of 7 randomized controlled trials and 15 observational studies including more than 140,000 patients treated with HD, found no evidence of increased risk of infection, cardiovascular events, hospitalizations, or mortality with the use of higher-dose IV iron compared with lower doses [32]. Together these findings suggest that higher doses of IV iron prescribed in the course of routine anemia management in HD patients may not be associated with substantial harm, although additional randomized trials will be needed to confirm these findings.

The strength of this study is that it included all prevalent patients in all DaVita HD facilities in two European countries that previously have not been part of the DOPPS analyses, thus representing a “real world” clinical experience of modern Western and Eastern European renal anemia practice in HD. Such real-world analyses allow us to evaluate existing paradigms of care and inform the design of new treatment options. Our study has several limitations. First, this was an observational, cross-sectional study design and was reliant on aggregated data: thus, we were unable to estimate the relative causal contributions of changes in IV iron or ESA dose to serum ferritin. Second, we were unable to make risk assessments for hospitalizations and mortality since we lack individual data on these issues. Third, we do not have data on accumulated iron doses over longer periods of time in each patient. An individual patient may, over time, be given a combination of low- or high-dose iron or ESA therapies based on monthly laboratory parameters such as Hb, TSAT and ferritin.

## Conclusions

In conclusion, the present international multicenter study analyzed clinical practices and treatment strategies to correct renal anemia in 1247 prevalent HD patients in 12 dialysis centers in two European countries that previously have not been included in the global DOPPS analyses. The KDIGO hemodialysis anemia target was reached in patients treated at the dialysis centers in both Portugal and Poland, however by two different treatment strategies. Since mortality was similar in the centers, our findings suggest that higher doses of IV iron prescribed for HD patients during in routine clinical practice may not be associated with significant harm. However, meticulously performed randomized controlled trials are needed to provide decisive evidence of iron safety.

## Abbreviations

AVF: Arteriovenous fistula
BMI: Body mass index
CVC: Central venous catheter
ESA: Erythropoiesis stimulating agent
ESRD: End-stage renal disease
Hb: Hemoglobin
HD: Hemodialysis
IV: Intravenous
PTH: Parathyroid hormone
SC: Subcutaneous
TSAT: Transferrin saturation
